# Perceived quality of life, 6 months after detoxification: Is abstinence a modifying factor?

**DOI:** 10.1007/s11136-016-1272-z

**Published:** 2016-03-19

**Authors:** John-Kåre Vederhus, Bente Birkeland, Thomas Clausen

**Affiliations:** 1 Addiction Unit, Sørlandet Hospital HF, P.b. 416, 4604 Kristiansand, Norway; 2 Norwegian Centre for Addiction Research, University of Oslo, Oslo, Norway

**Keywords:** Substance use disorders, Treatment outcome, Norway

## Abstract

**Purpose:**

Patients with a substance use disorder (SUD), admitted for detoxification, often suffer from a poor quality of life (QoL). We set out to monitor QoL, together with substance use, in a departure from the usual norm of measuring substance use alone as a treatment outcome. Literature searches revealed scant knowledge of how QoL is influenced. With this in mind, we aimed to investigate whether total abstinence, prior to follow-up, could influence QoL.

**Methods:**

We studied a prospective cohort of 140 patients admitted for inpatient detoxification treatment at Sørlandet Hospital (Norway), from September 2008 to August 2010. QoL was measured by a generic five-item questionnaire, the QoL-5. The extremes of this scale ranged from the worst possible rating of 0.1 to 0.9, as the best. A norm for the general population was benchmarked at 0.69. Change in QoL was calculated by subtracting baseline QoL from that achieved at the 6-month follow-up interview; linear regression modeling was used to study the influence of individual QoL predictors.

**Results:**

The mean QoL at baseline was 0.46, 39 % below that of the general reference population. By applying the clinical interpretation of the scale, we found a modest overall mean improvement in QoL at follow-up (0.11 points); the greatest increases were seen for patients with the lowest baseline QoL scores. Abstinence prior to follow-up correlated with improved QoL, while living alone and psychological distress were negative influences.

**Conclusions:**

For patients with a SUD, clinicians should emphasize that abstinence may help to improve their QoL.

## Background

Substance use disorders (SUDs) cause a spectrum of health problems, one of which is to increase the number of years lived with a disability [1]. Research in this area indicates that a SUD can affect well-being and function across a number of areas in life, and may lead to a considerable deterioration of physical health [2, 3], and social functioning [4]. Psychiatric disorders and SUDs are also common comorbidities; thus, impaired psychological health and reduced well-being often co-exist [5]. Physical health and mental health are the two integral quality of life (QoL) components [6]. For SUD researchers, their evaluation represents a qualitative improvement in outcome measurement, beyond the established practice of focusing solely on substance use [7]. The addiction research field has, to some degree, responded to the need to investigate QoL more methodically, and the number of articles that derive correlates between QoL and substance use has increased in the last decade [3, 8, 9].

The goal of SUD treatment is to initiate rehabilitation, promote continued abstinence, or at least reduce substance use and to help patients to become engaged with their own long-term recovery [10, 11]. An overarching aim is therefore to improve QoL. If living without substance use is to be the goal for the SUD patient, then this outcome must be associated with improved well-being, for at least some facets of life. Otherwise, this aim will simply not be perceived as “worth” fighting for. Consequently, patients may lose motivation and interest in their rehabilitative process [12].

Detoxification (detox) treatment is considered to be one way to initiate the recovery process. Ideally, patients should transition post-detox, to more extensive and longer term SUD treatment. The reality though is that many patients will only experience detox [13]. As a stand-alone intervention, detox may fail to prevent the patient from reverting back to a state of continued substance use [13].

The QoL of patients admitted to detox has, to some degree, been assessed, but many studies only utilize cross-sectional designs, survey patients only at admission and discharge [14–16], or typically have short follow-up periods, e.g., up to 3 months [17]. Thus, there is still a lack of prospective studies that have examined QoL changes at lengthier time frames after detox. Encouraging exceptions exist though. Picci et al. [18] examined variations in QoL, up to 12 months following detox, to evaluate the predictive value of QoL for relapse and the severity of alcohol use. They found that baseline QoL was not predictive of either relapse or alcohol use severity at follow-up. A secondary finding was that a significant QoL improvement was seen among patients who had achieved abstinence, with QoL scores among relapsed patients unchanged.

This finding epitomizes a typical dispute in this field: whether abstinence is a necessary precondition for QoL improvement or whether any reduction in substance use will, as a byproduct, improve QoL [7, 19]. As previously mentioned, there is evidence to show that QoL improves with abstinence and deteriorates with relapse [18, 20], but the findings are mixed. For example, a recent Norwegian study found no association between abstinence and QoL among SUD patients at their six-month post-treatment follow-up [21]. In a second, US-based study, three months of outpatient SUD treatment also failed to reveal any correlates between QoL and changes in alcohol and drug consumption [22]. Similarly, in a series of shorter detox studies, Foster et al. [17, 23, 24] reported an improvement in QoL for only some alcohol-dependent individuals when they changed their pattern of alcohol usage. The question has also been raised as to the definition of the rather broad term “recovery”. Is abstinence a defining element, or is it just one of many strategies for achieving recovery [10, 25]. The present study is among the few studies focusing on QoL after detox treatment that have a longer follow-up than 3 months and have the relationship between QoL and abstinence as its main focus.

### Purpose

The aim of this study was to (1) examine QoL changes from baseline to the six-month follow-up interview (post-discharge) and (2) determine whether abstinence in the month preceding the follow-up interview predicted outcome (QoL), while controlling for other potential variables. We hypothesized that being abstinent during the 30 days preceding the follow-up interview would positively predict improved QoL.

## Methods

### Study setting

This study reports pre- and post-treatment QoL status of a cohort of patients recruited to a controlled trial on a detoxification ward at the Addiction Unit, Sørlandet Hospital, in Kristiansand, Norway, between September 2008 and August 2010. Detoxification treatment in Norway typically engages three types of patient group; patients detoxed before their admittance into longer term, inpatient treatment; patients initiating opioid maintenance treatment (OMT); and patients who receive a detox and are subsequently discharged. The latter group, those discharged with no immediate plans for further inpatient or OMT treatment, was approached to enroll in this study. Their eligibility was based on the central tenet of this study, to test how patients, post-discharge, could be motivated to seek their own support in community-based, addiction-related, mutual-help groups (MHGs) [11]. Exclusion criteria for our study included severe psychiatric disorders or cognitive impairment. Of 156 eligible patients, 16 declined to participate, leaving a final cohort of 140 patients, representing 89 % of the original eligible respondents (Table 1). We have previously published a detailed description of our patient cohort, and their treatment setting, in the context of a separate study on motivational intervention [11]. The Regional Ethics Committee of the South-East Health Region, Norway, approved the study.

**Table 1:** Characteristics of study respondents (*N* = 140)

Characteristic	N (%) or *mean (SD)*
Age, years	*41* (*14*)
Female	45 (32)
Proportion native Norwegians or European origin	134 (96)
Education, years	*11.2* (*2.3*)
Relationship, proportion living in relationship	74 (53)
Main diagnosis (ICD-10)	
(1) Alcohol dependence (*N* = 48) or harmful alcohol use (*N* = 6)	54 (39)
(2) Both alcohol and drug dependence	26 (19)
(3) Drug dependence	60 (43)
Severity variables	
Earlier SUD treatment (prior to current detoxification)	108 (77)
Years of problematic use^a^ of the major drugs of abuse	*11.4* (*9.0*)
Alcohol composite score (EuropASI)^b^	*0.43* (*0.36*)
Drug composite score (EuropASI)^b^	*0.25* (*0.20*)
Self-rated substance use severity^c^	*4.2* (*0.7*)
Injection use in the last 6 months	40 (29)
Psychological distress^d^	*2.4* (*0.7*)
Quality of life^e^	*0.46* (*0.15*)
Days on the ward	*11* (*5*)

^a^Problematic use, as defined in EuropASI, was the consumption of 5 or more standard drinks at least 3 times weekly, or binge drinking on 2 consecutive days to a level that affected daily functioning. For drug use, only frequency was needed; 3 times weekly or 2 consecutive days

^b^EuropASI composite score, scale 0–1

^c^SYRAAP, severity score of the substance use, scale 1–5

^d^SCL-10, global score index, scale 1–4

^e^QoL-5, scale 0.1–0.9

### Measures and procedures

To avoid any influence of withdrawal symptoms on QoL baseline scores, patients were neither approached nor recruited to this study until they had passed the acute detoxification phase. Therefore, patients were first approached to participate in this study at a mean timepoint of 4.5 days after their admission. After providing informed consent, participants were assessed with a QoL measure (see below) and completed the semi-structured EuropASI interview to collect data on patient demographics, background, treatment history, and substance use [26]. The time frame for the EuropASI is the 30 days preceding the interview, and data on drug and alcohol use in the 30 days prior to admission yielded composite scores to indicate severity of substance abuse [27]. Scores ranged from 0 (no problem) to 1 (a severe problem). As the ward admitted patients with both alcohol and drug dependence, we also included an overall substance use severity measure; the Survey of Readiness for AA Participation (SYRAAP) severity subscale [28]. The five questions of the scale, e.g., “My substance use has hurt some other people” and “Using substances has interfered with my ability to deal with everyday problems,” were rated on a five-point Likert-type response format, from scale 1 (strongly disagree) to scale 5 (strongly agree). A mean score was computed; a higher score meant higher severity. A score ≥4 on the SYRAAP scale is considered to reflect a serious substance use problem [29]. The Mini International Neuropsychiatric Interview (MINI), version 5.0, was used to confirm the SUD diagnosis [27]. To assess mental health, we used Symptom Check List-10 (SCL-10), a measure of psychological distress (scale 1–4 [30]). A mean score (global score index) was computed; the higher the score, the greater the distress. A score of ≥1.85 using SCL-10 is considered to be a pathological score [29, 30].

At the six-month follow-up interview, 113 patients (81 %) were successfully contacted and re-assessed using Europ-ASI and the QoL survey described below. Those lost to follow-up were younger (35 vs. 43 years; *t* = 2.6, degrees of freedom = 138; *p* < 0.01), but otherwise had no defining characteristics. The mean QoL scores at baseline for those lost to follow-up were comparable to those who engaged with the study.

#### Outcome

Quality of life was measured at baseline and at follow-up using the QoL-5 test, a short, generic QoL instrument. This survey does not focus on any disease-related deficits, but instead assesses the patient’s satisfaction with life in general [31]. QoL-5 consists of five subjective statements: two questions are about health, physical, and mental; two questions address the quality of significant relationships (partner and friends); and one question addresses the existential self, i.e., the relationship with oneself. Responses were scored on a five-step ordinal scale from 1 to 5. A score of 1 is very good, and 5, very poor. The raw scores were then transposed, and inverted as a decimal scale ranging from 0.1 to 0.9; 0.9 was now the best score, and 0.1, the worst [32]. Mean scores for health, relationships, and the existential self, were calculated, and a total QoL score, derived. For patients without a partner, the relationship sub-score was based on one question only. Normative data from a previous survey of the general population showed a mean QoL score of 0.69 [31, personal communication]. This was used as our reference population QoL. The cutoff score for a markedly reduced QoL was suggested to be ~0.15 below that of the general population (≤0.55). Scores lower than 0.40 were considered to be severely reduced [21]. Changes in QoL were computed by subtracting the QoL determined at admission from the QoL obtained at follow-up, hereafter called the “QoL score change”. A QoL score increase from baseline to follow-up of 0.2 (a one-point increase on the raw score scale, e.g., from “good” to “very good”) or higher was denoted as substantial and indicated a clinically important improvement. Other QoL changes were considered moderate (≥0.1 score), small (≥0.05 score), or very small (<0.05) [31, 32]. The internal consistency of the scale was good; the Cronbach’s alpha coefficient was 0.75 and 0.81 for the QoL-5 at baseline and follow-up, respectively [33].

#### Factors associated with QoL

To examine determinants of QoL at follow-up, the socio-demographic variables of gender, age, and whether patients were in a relationship at baseline were included as potential predictors. Of the clinical variables collected at baseline, we included patients’ self-rated severity of substance use using the SYRAAP severity score and their perceived mental health status using the SCL-10 assessment. The reliability of the scales was good; the Cronbach’s alpha coefficient was 0.75 and 0.91 for the SYRAAP and SCL-10, respectively. We also took into account two additional predictors that could influence outcome: inpatient treatment (scored in days) and MHG support (scored as number of MHG meetings attended) during the six-month follow-up period. Substance use at follow-up was assessed with the EuropASI. Substance use status and whether patients were abstinent or not was determined according to self-reported alcohol and drug use for the 30 days preceding the follow-up interview, i.e., the abstinent group had no alcohol or drug use during this period.

### Statistical analyses

Descriptive statistics were used to elaborate baseline characteristics. To descriptively show the 6-month change in relation to patients’ QoL status, QoL changes were grouped according to the baseline QoL status. These were either severely reduced (<0.40), markedly reduced (0.40–0.55), or close to normal QoL (>0.55). Two different *t*-tests were used; paired sample *t*-test was used to examine the 6-month change in substance use and QoL, and the Student’s *t*-test was used to explore between group differences on the QoL change score and QoL score at follow-up.

Linear regression with simultaneous entry of variables (the “enter” method) was used to examine predictors of QoL at follow-up. Baseline QoL was included in the analysis to control for a possible ceiling effect, i.e., those who already had a near-to-normal QoL at baseline were not expected to manifest any marked improvement. Results are presented as unstandardized beta coefficients with 95 % confidence interval (CI). The *R*-square (*R*^2^) value was used to assess the proportion of variability in the dataset. Analyses of variables were considered to be statistically significant at a *p* value of <0.05; all analyses were performed using IBM SPSS Statistics version 21.

## Results

The sample was a mixed population with either an alcohol and/or a drug use disorder. Patients had in excess of 11 years of problematic use of their major substance of abuse; 77 % had received prior specialized SUD treatment, and 29 % had used an injected drug in the 6 months prior to admission (Table 1). The mean QoL for our cohort at baseline was 0.46, which is 0.23 below that of the general population, representing a relative drop of 39 % when taking the lowest extreme of the scale into account. Thirty-seven percent of our patients had a markedly reduced QoL score (0.40–0.55), and a further 32 % had a severely reduced QoL (<0.40).

Although the patients were not intended to be directly transferred to further SUD treatment upon discharge, those who were reached at follow-up had received a mean of 18 days (median 0, range 0–180) of inpatient treatment during the 6-month follow-up period. They had also attended a mean of 12 MHG meetings (median 1, range 0–97). Patients reported a significant reduction in substance use; the alcohol composite score using the EuropASI test decreased from 0.45 to 0.21 (−0.24, 95 % CI for the difference = −0.18/−0.31, *p* < 0.001), and the drug composite score decreased from 0.25 to 0.10 (−0.14, 95 % CI for the difference = −0.11/−0.17, *p* < 0.001). Almost half of the sample, 52 patients (46 %), reported total abstinence from all substances for the 30 days preceding the follow-up interview.

**Fig. 1:**
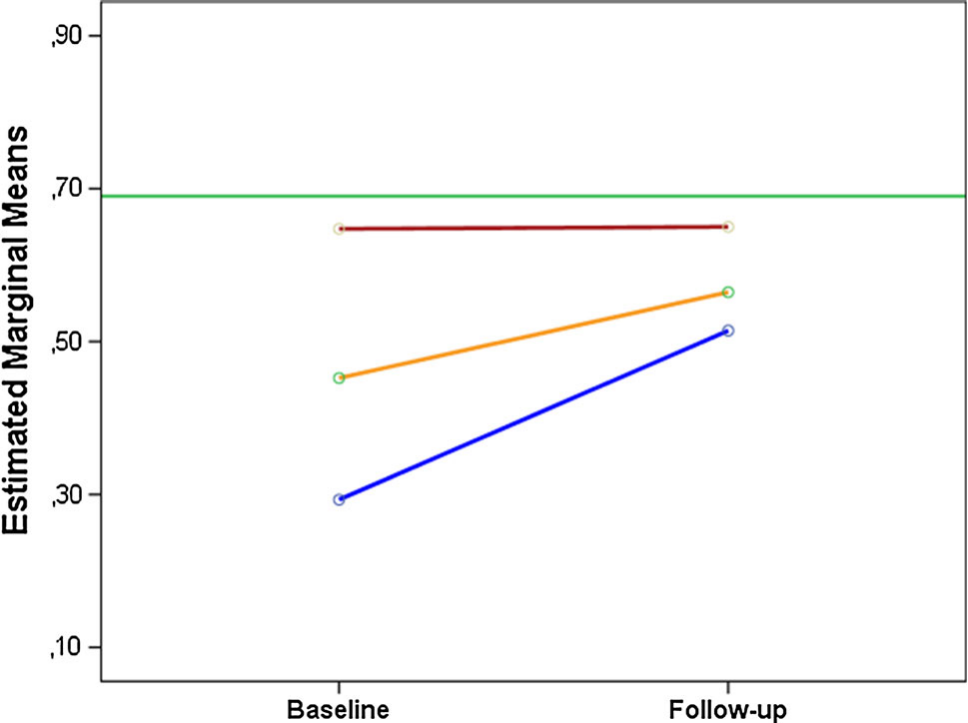
QoL changes from baseline to follow-up based on QoL status at baseline (*N* = 113). *Green line* mean score of a general reference population. *Red line* mean change of patients with near-to-normal QoL at baseline (>0.55). *Yellow line* mean change of patients with markedly reduced QoL at baseline (0.40–0.55). *Blue line* mean change of patients with severely reduced QoL at baseline (<0.40)

**Fig. 2:**
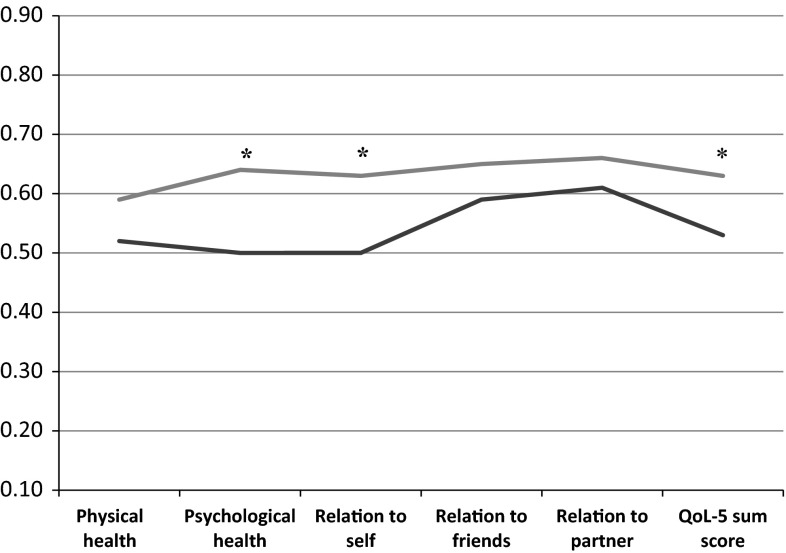
Comparison of QoL scores of patients that were abstinent or relapsed at follow-up (*N* = 113). *Blue line* patients abstinent last 30 days before follow-up. *Red line* patients relapsed in the last 30 days before follow-up. *p value for the difference (Student’s *t*-test) < 0.05

In terms of QoL change, for the sample as a whole, there was only a modest improvement (0.11, 95 % CI 0.08–0.15, *p* < 0.001, paired sample *t*-test), contributed to in the main by more pronounced progress among those with the lowest baseline scores (Fig. 1 ). With reference to the main focus of this study, whether abstinence status was associated with differences in QoL, those who were abstinent before follow-up recorded substantial, and clinically relevant, improvements (0.19, 95 % CI 0.13–0.25). In comparison, those reporting continued substance use achieved small improvements in QoL (0.05, 95 % CI 0.01–0.08). Thus, the unadjusted mean difference between the groups was 0.14 and was found to be statistically significant (95 % CI 0.08–0.21, *p* < 0.001). Additionally, the two groups were assessed by a 6-month, post-treatment survey, for their mean scores for each individual QoL item. Collectively, abstinence prior to follow-up resulted in improved scores for psychological health, existential QoL, and overall QoL-5 (Fig. 2).

Considering abstinence as a predictor for QoL in a multiple linear model, and when controlling for demographic and clinical variables, abstinence was still found to be a significant predictor (*β* 0.12, 95 % CI 0.06–0.18, Table 2). The unadjusted significance of abstinence on QoL was slightly greater (0.14). Other significant predictors were living alone (*β* −0.08, 95 % CI −0.14/−0.02) and psychological distress (*β* −0.08, 95 % CI −0.13/−0.02); these both influenced QoL negatively. Our analysis was adjusted for the QoL-5 score at baseline, which in itself was not significant. Neither inpatient treatment, nor MHG attendance during follow-up, contributed to any variance for our dependent variable. In terms of data fit, our model explained 33 % (*R*^2^) of the variance found for QoL.

**Table 2:** Predictors of QoL at follow-up (*N* = 113)

Predictor	*β* (95 % CI)^a^	*p* value
Socio-demographic variables		
Gender (female)	0.00 (−0.07/0.05)	0.844
Age, years	0.00 (−0.00/0.00)	0.921
Living alone	−0.08 (−0.14/−0.02)	0.007
Psychological distress^c^	−0.08 (−0.13/−0.02)	0.007
Self-rated substance use severity^b^	0.00 (−0.04/0.06)	0.796
QoL-5 at baseline	0.19 (−0.05/0.43)	0.114
Follow-up variables		
Mutual-help group participation^d^	0.00 (−0.00/0.00)	0.759
Inpatient SUD treatment during follow-up (days)	0.00 (−0.00/0.00)	0.779
Abstinence in the 30 days before follow-up	0.12 (0.06/0.18)	<0.001

^a^Multiple linear regression with simultaneous entry of variables (the “enter” method); unstandardized beta coefficient with 95 % confidence interval (CI)

^b^Perceived severity of the substance use, a subscale of the Survey of Readiness for AA Participation; SYRAAP

^c^Symptom Check List-10, global score index

^d^Number of meetings in addiction-related mutual-help groups during follow-up

## Discussion

The majority of patients with SUDs undergoing detoxification had markedly impaired QoL at treatment inclusion. At the six-month follow-up, those who were abstinent had a substantial improvement in their QoL, while those still using substances manifested a modest improvement in QoL. Multivariate analysis corroborated these findings, with abstinence identified as a positive predictor of QoL, while living alone and psychological distress were negative influences.

The markedly reduced QoL of SUD patients at admission confirms findings in previous studies that patients with SUDs experience low levels of QoL compared with the general population and compared to those with other chronic health conditions [34]. This has also been seen in previous samples of patients admitted for detox [15, 35]. For example, compared to the normative score for the general population, a ~22 % reduction in score was found using the SF-36 survey, for patients on their admission to detoxification in a French study [15]. The mean QoL score in the present study was even lower, 39 % lower than that of the reference population. We should, however, note one caveat for this comparison that different measures were used to assess QoL. Therefore, the French study [15] and the current study are not directly comparable.

Considering QoL change, there appeared to be a ceiling effect in that those patients with a near-normal QoL at baseline had unchanged QoL at follow-up; any substantial improvement was confined to those who were initially worse off. This has previously been shown in a study that measured QoL during a short residential stay of 3 weeks [15]. Our study, with its 6-month follow-up interview, showed that a higher QoL at baseline was not predictive of a superior QoL at the 6-month after discharge. This is somewhat contrary to findings in the large New European Alcoholism Treatment study (NEAT), in which the patients with an initially poor QoL had a worse prognosis [35] However, although those with markedly reduced and severely reduced QoL at baseline achieved the most pronounced progress in the current study, they still failed to attain the QoL scores of those who had a near-normal QoL score at baseline.

As hypothesized, abstinence was associated with a better QoL at follow-up, similar to the findings of Picci et al. [18]. Unlike that study, however, our non-abstinent participants also reported some improvement in their QoL, but this was considered to be a minor influence when evaluated clinically. Thus, our study indicates that achieving abstinence is an important factor in improving QoL. Both abstinent and non-abstinent patients rated the quality of their relationships to friends and partners at a similar level, but the groups differed significantly in their psychological health, and relationship to self, assessments. Broadly put, the improved QoL of those who were abstinent seemed to be brought about by improved psychological health and a positive change in their relationship to self. This indicates that individual components of QoL change differently with time in relation to recovery status and that abstinence predominantly influenced these two QoL components. Thus, with abstinence, there are parallel gains in emotional and ontological health.

Living alone, i.e., living without support from a close partner resulted in a deterioration in QoL. Patients with a SUD often suffer from broken relationships or the family and/or social network may be worn out by trying to help or mitigate the consequences of the condition [36, 37]. Hence, in the case of a patient with a SUD, living alone may not necessarily be a choice. The upshot is that positive familial restraining influences may no longer be present. There may also be a lack of motivational support to promote self-help and agency in the patient. Our data agree with an earlier study among alcoholics undergoing rehabilitation [38]. In that case, the authors found that the perception of being lonely and feelings of loneliness were robust predictors of poor QoL and prognosis.

A previous meta-analysis found that the most powerful predictor of lower levels of QoL at admission to SUD treatment was the perceived severity of substance abuse [34]. Concerning variables predictive of QoL at study end, the large longitudinal NEAT study found that substance use severity at baseline did not negatively influence QoL at follow-up [35]. We reached a similar conclusion, finding that substance use severity at treatment admission did not lead to a worse prognosis in terms of perceived QoL at a later stage. On the other hand, distress in the mental health domain was a potent negative predictor of QoL at follow-up, as previously found in a study of outpatient participants, in which better mental health at baseline predicted better QoL at a 3-month follow-up [22]. These findings imply that patients with a greater psychiatric deficit are inherently disadvantaged when it comes to improving their QoL.

Unexpectedly, neither inpatient SUD treatment nor MHG support positively influenced QoL at follow-up. However, our mean values for these data may have been skewed by the fact that the majority of our patients had no inpatient treatment and attended less than two MHG meeting during the six months, as evidenced by the median values for these variables.

### Methodological considerations

The strength of the study is its use of a prospective design that allows the examination of QoL change with time. Our findings must, however, be interpreted in the context of certain study limitations such as a moderate sample size. Substance use outcomes were only taken into account for the 30 days preceding the follow-up interview, with no data for the first 5 months after discharge. Thus, we were not able to evaluate whether the duration of abstinence influenced outcome, which has been mentioned as an important factor for any increase in QoL following treatment [7, 35].

## Implications

Measuring outcome following SUD treatment should not only be based on a traditional assessment of SUD symptoms and SUD-related problems, but instead should incorporate measurements of global health. This innovation would allow us to examine whether a reduction in SUD symptoms occurs contemporaneously with improved physical, emotional, relational, and ontological health [10]. Thus, measurements of QoL should be used to complement traditional outcome assessments. This would help to emphasize and personalize the patient’s own experience and perception of their illness and promote a greater awareness of the value of QoL outcomes following therapy. It would also help the clinical field to shift to a more solution-focused recovery paradigm. Although our findings indicate that abstinence from substance usage is important, rehabilitation should be seen as a more complex process than simply an altered pattern of substance use [10].

## Conclusions

SUD populations admitted for inpatient detox treatment suffer from poor QoL at admission. Improvements in QoL were hampered by psychological distress and by living without close support, but were enhanced by total abstinence. Patients should be encouraged to obtain abstinence-oriented support, e.g., from formal treatment and/or addiction-related mutual aid groups, in order to maximize QoL improvements. Treatment providers need to address the patient’s psychological status, implement strategies to improve social function, and thereby promote the patient’s interest and inclination to seek further help and support.
